# Tyrosine-Kinase Inhibitors Therapies with Mainly Anti-Angiogenic Activity in Advanced Renal Cell Carcinoma: Value of PET/CT in Response Evaluation

**DOI:** 10.3390/ijms18091937

**Published:** 2017-09-09

**Authors:** Girolamo Ranieri, Ilaria Marech, Artor Niccoli Asabella, Alessandra Di Palo, Mariangela Porcelli, Valentina Lavelli, Giuseppe Rubini, Cristina Ferrari, Cosmo Damiano Gadaleta

**Affiliations:** 1 Interventional Radiology Unit with Integrated Section of Medical Oncology, National Cancer Research Centre, Istituto Tumori “Giovanni Paolo II”, Bary 70124, Italy; ilariamare@tin.it (I.M.); dipaloalessandra@gmail.com (A.D.P.); marypor@libero.it (M.P.); ferrari_cristina@inwind.it (C.F.); cd.gadaleta@gmail.com (C.D.G.); 2 Nuclear Medicine Unit, University of Bari “Aldo Moro”, Bari 70124, Italy; artor.niccoliasabella@uniba.it (A.N.A.); valentina.lavelli@gmail.com (V.L.); giuseppe.rubini@uniba.it (G.R.)

**Keywords:** renal cell carcinoma, tumor angiogenesis, tyrosine kinase inhibitors, tyrosine kinase receptor, PET/CT, ^18^F-FDG, other radiotracers

## Abstract

Renal cell carcinoma (RCC) is the most frequent renal tumor and the majority of patients are diagnosed with advanced disease. Tumor angiogenesis plays a crucial role in the development and progression of RCC together with hypoxia and glucose metabolism. These three pathways are strictly connected to the cell growth and proliferation, like a loop that is self-feeding. Over the last few years, the ever-deeper knowledge of its contribution in metastatic RCC led to the discovery of numerous tyrosine kinase inhibitors (TKIs) targeting pro-angiogenic receptors at different levels such as sunitinib, sorafenib, pazopanib, axitinib, tivozanib, and dovitinib. As anti-angiogenic agents, TKIs interfere the loop, being able to inhibit tumor proliferation. TKIs are now available treatments for advanced RCC, which demonstrated to improve overall survival and/or progression free survival. Their effects can be detectable early on Positron Emission Tomography/Computed Tomography (PET/CT) by change in ^18^F-fluoro-2-deoxy-2-d-glucose (^18^F-FDG) uptake, the main radiotracer used to date, as a strong indicator of biological response. ^18^F-FDG PET/CT demonstrated an ability to predict and monitor disease progression, allowing an early and reliable identification of responders, and could be used for image-guided optimization and “personalization” of anti-angiogenic regimens. New radiotracers for biometabolic imaging are currently under investigation, which exploit the other pathways involved in the cancer process, including cellular proliferation, aerobic metabolism, cell membrane synthesis, hypoxia and amino acid transport, as well as the angiogenic process, but they require further studies.

## 1. Introduction

### 1.1. Background

**Figure 1:**
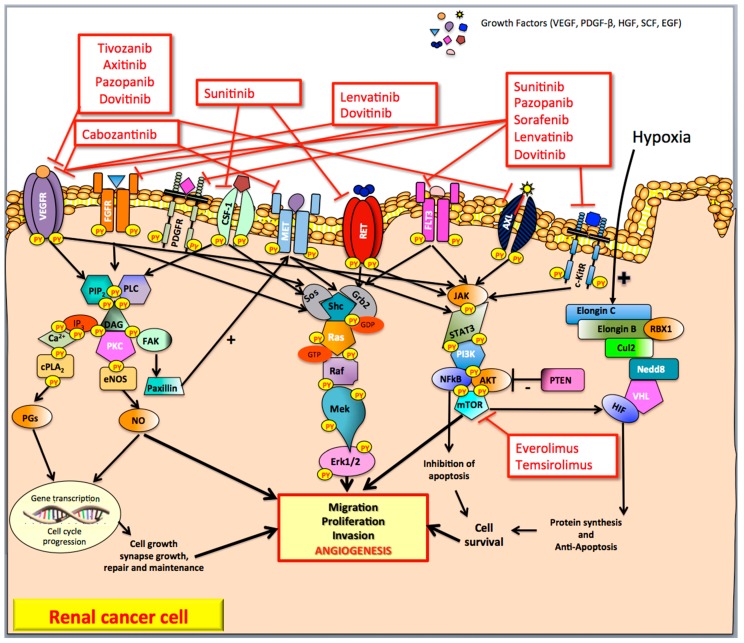
Pathways and tyrosine kinase inhibitors targeting angiogenesis in advanced clear-cell renal carcinoma. In renal cancer, numerous cell growth factors binding several trans-membrane tyrosine kinase receptors (such as VEGFR, FGFR, PDGFR, HGFR) activate the RAS GTPase proteins. Protein kinase C, RAS-MAP kinase and phosphoinositide 3-kinase are the pathways commonly involved in renal cancer cell (as well as in endothelial and stromal cells) development leading mainly to tumor angiogenesis. In addition, hypoxia induces hypoxia-inducible factor to stimulate the transcription of genes that lead to the secretion of pro-angiogenic factors. VEGF, Vascular Endothelial Growth Factor; PDGF-β, Platelet Derived Growth Factor-β; HGF, Hepatocyte Growth Factor; SCF, Stem Cell Factor, EGF; Epidermal Growth Factor; VEGFR, Vascular Endothelial Growth Factor Receptor; FGFR, Fibroblast Growth Factor Receptor; PDGFR, Platelet Derived Growth Factor Receptor; CSF1-R, Colony Stimulating Factor 1 Receptor; MET, Mesenchymal-Epithelial Transition factor or Hepatocyte Growth Factor Receptor; RET-R, REarranged during Transfection Receptor; FLT-3, FMS-like tyrosine kinase 3; AXL, AXL Receptor; c-KitR, c-Kit Receptor; PIP_2_, Phospholipid phosphatidylinositol 4,5-bisphosphate; PLC, Phospholipase C; IP3, Inositol 1,4,5-trisphosphate; DAG, Diacylglycerol; PKC, Protein Kinase C; FAK, Focal Adhesion Kinase; cPLA2, Phospholipase A2; PGs, Prostaglandins; eNOS, endothelial Nitric Oxide NO synthase; NO, Nitric Oxide; Sos, Son of sevenless; Grb2, Growth factor receptor-bound protein 2; Shc, SHC-adaptor protein; Ras, Rat sarcoma protein; Raf, RAF proto-oncogene serine/threonine-protein kinase; Mek1/2, MAP kinase-ERK kinase; Erk1/2, Elk-related tyrosine kinase; JAK, Janus kinase; STAT3, signal transducer and activator of transcription 3; PI3K, phosphatidyl inositol 3-kinase; NFkB, nuclear factor kappa B; AKT, protein kinase B; mTOR, mammalian Target Of Rapamycin; PTEN, Phosphatase and TEnsiN homolog; VHL, von Hippel–Lindau protein; Cul2, cullin2, Nedd8, neural precursor cell expressed developmentally down-regulated 8; RBX1, ring-box 1; VEC, E3 ubiquitin ligase complex; HIF, hypoxia-inducible factor; +, positive feedback (stimulate); −, negative feedback (inhibit).

Renal cell carcinoma (RCC) is the most frequent renal tumor, accounting for 3% of all adult cancers [1]. Approximately 50–70% of patients are diagnosed with advanced disease and with clear-cell (cc) histotype [2]. It is well known that tumor angiogenesis plays a crucial role in the etiopathogenesis of cc-RCC [3].

cc-RCC is characterized by the inactivation of the von Hippel-Lindau (VHL) tumor suppressor gene that segregates in the chromosomal region 3 (p25–26) [4]. The chromosomal alteration of the VHL tumor suppressor gene leads to the functional inactivation (due to the loss of both alleles) of the von Hippel-Lindau protein (pVHL), an essential component of the cellular oxygen-sensing pathway. The functional form of the pVHL, in association with elongin C, elongin B, cullin2 (Cul2), neural precursor cell expressed developmentally down-regulated 8 (Nedd8) and ring-box 1 (RBX1), forms a multiprotein complex called E3 ubiquitin ligase (or VEC) able in turn to bind the hydroxylated form of the subunit α of the transcription hypoxia-inducible factor (HIF) [5,6,7]. In normoxic conditions, the formation of this complex leads to the degradation of HIF, while, in the case of hypoxia, the stabilized form (non-hydroxylated) HIF is able to induce the transcription of genes that lead to the secretion of pro-angiogenic growth factors (GFs) such as Vascular Endothelial Growth Factor (VEGF), Fibroblast Growth Factor-2 (FGF-2), Platelet Derived Growth Factor-β (PDGF-β), Hepatocyte Growth Factor (HGF), and Stem Cell Factor (SCF) [3,8] (Figure 1).

GFs bind several receptors (such as VEGFR, FGFR, PDGFR, and HGFR), belonging to the class III of trans-membrane tyrosine kinase (TK) receptors that, in turn, activate the RAS GTPase proteins (Figure 1). Protein kinase C (PKC), RAS-MAP kinase and phosphoinositide 3-kinase (PI3K) are the upregulated pathways commonly involved in cc-RCC development [3,9] (Figure 1).

The hydrolysis of phospholipid phosphatidylinositol 4,5-bisphosphate (PIP2) by phospholipase C (PLC) produces two distinct second messengers, diacylglycerol (DAG) and inositol 1,4,5-trisphosphate (IP3) [9]. DAG and IP3 stimulate distinct downstream signaling pathways (PKC and Ca^2+^ mobilization, respectively). IP3 induces a cytosolic Ca^2+^ levels increase, which affects the activities of a variety of target PKs and phosphatases (such as phospholipase A_2_, and cPLA_2_ that induces the secretion of prostaglandins (PGs)) leading to transcription factor phosphorylation, changes in gene expression, and stimulation of cell proliferation. PKC pathway is also involved in the upregulation of endothelial NO synthase (eNOS) that leads to the increased production of nitric oxide (NO) [10]. NO promotes tumor angiogenesis directly mediating the recruitment of perivascular cells and, therefore, remodeling and maturation of blood vessels. The phosphorylation of focal adhesion kinase (FAK) regulates the interaction of FAK with paxillin, a protein that plays a crucial role in the upregulation of MET TK signaling pathway in RCC [11].

The main RAS effector pathway is the RAF-mitogen-activated protein (MAP)/extracellular signal-regulated kinase (ERK) kinase [12]. Once RAF proteins are activated and phosphorylated by different protein kinases, they phosphorylate MEK, which in turn activates ERK-1 and ERK-2 [13]. This network modulates gene expression via the phosphorylation of transcription factors with profound effects on tumorigenesis, mainly mediated by angiogenesis.

The RAS-MAPK pathway is a fundamental controlling of the phosphoinositide-3-kinase PI3K/AKT/mTOR (mammalian target of rapamycin) pathway. This pathway induces the Janus kinase (JAK)/signal transducer and the (STAT) pathway, which represents a central regulator of the proliferation of RCCs. The PI3K/AKT/mTOR pathway is activated in RCC and its blockade by rapamycin and/or everolimus and temsirolimus has been shown to inhibit the growth of RCC [14].

Up to now, some progress in the treatment of RCC such as immunotherapy and radiotherapy has been made. This is particularly true in the field of immunotherapy for the introduction in clinical practice of Nivolumab, the only approved anti-programmed cell death-1 immune checkpoint inhibitor [15]. In addition, radiotherapy, with special references to the stereotactic ablative radiotherapy, which allows the delivery of high doses on small treatment volumes, was applied for the treatment of the primary and oligometastasic RCC, obtaining interesting results [16].

However, the ever-deeper knowledge on molecular biology of advanced cc-RCC has led to the discovery of numerous tyrosine kinase inhibitors (TKIs) targeting specifically tumor angiogenesis: sunitinib, pazopanib, sorafenib, axitinib, cabozantinib, dovitinib, lenvatinib and tivozanib. Multi-targeted TKIs are systematic treatments available for advanced cc-RCC targeting improving patients’ overall survival (OS) and/or progression free survival (PFS) [3,17]. The antitumor activity of TKIs is not cytotoxic like classical antitumor therapeutics, but rather cytostatic, suppressing biological activity by inhibiting tumor angiogenesis. Practically, some cc-RCC treated with the TKIs does not decrease in tumor volume but enters a period of long-term dormancy, without enlargement of volume or novel metastasis. It has been suggested that a new assessment focusing not only on the volume of the tumors, but also biological activities to evaluate the antitumor activity of TKIs is necessary.

### 1.2. Tyrosine Kinase Inhibitors (TKIs) Targeting Angiogenesis

Sunitinib is an oral multi-targeted receptor TKI, which binds to VEGFR-1/2/3, PDGFRα/β, c-KitR, FLT-3, CSF1-R and RET-R [18]. In the pivotal phase III trial, Motzer et al. have evaluated sunitinib (at a dose of 50 mg daily, four weeks on and two week off) compared to interferon-α2a (IFN-α, nine MIU triweekly) in 750 patients with advanced RCC as first-line treatment [19]. The study has demonstrated a statistically significant improvement in median PFS in patients receiving sunitinib (11 months) compared to patients receiving IFN-α (five months) (*p* < 0.001). The overall response rate (ORR) was significantly superior in sunitinib subgroup (47%) compared to placebo subgroup (12%) (*p* = 0.001). It has been observed statistically significant difference in terms of OS between two treatment arms (median OS of 26.4 months in sunitinib arm versus 20 months in sorafenib arm; *p* = 0.036). Based on these results, sunitinib (Sutent^®^, Pfizer Inc., New York, NY, USA) was approved in March 2007 by the Food and Drug Administration (FDA) and in August 2006 by the European Medicines Agency (EMA) for the treatment of advanced RCC.

Pazopanib is an oral multi-targeted receptor TKI, which binds to VEGFR-1/2/3, PDGFR-α/β, FGFR-1/2, and c-KitR [20]. In the pivotal phase III trial, Sternberg et al. have evaluated pazopanib (at a dose of 800 mg daily) compared to placebo in 435 advanced RCC patients as first-line treatment (54%) or whose disease had progressed on one previous systemic therapy with cytokines (46%) [21]. The study has demonstrated a statistically significant improvement in median PFS in patients receiving pazopanib (9.2 months) compared to patients receiving placebo (4.2 months) (*p* < 0.0001). Interestingly, in treatment-naïve subpopulation, the median PFS was longer in patients treated with pazopanib (11.1 months) than in patients treated with placebo (2.8 months) (*p* = 0.001). No significant difference in terms of OS between patients in both treatment arms (median OS of 22.9 months in pazopanib arm versus 20.5 months in sorafenib arm; *p* = 0.224) has been observed. Based on these results, pazopanib (Votrient^®^, GlaxoSmithKline, Research Triangle Park, North Carolina, NC, USA) was approved in October 2009 by the FDA and in June 2010 by the EMA for the treatment of advanced RCC.

Sorafenib is an oral multi-targeted receptor TKI that binds to BRAF, VEGFR-2, PDGFR, FLT-3 and c-KitR [22]. In the pivotal phase III trial TARGET study, sorafenib (at a dose of 400 mg bid daily) has been evaluated compared to placebo in 795 advanced RCC patients as first-line treatment (20%) or whose disease had progressed on one previous systemic therapy with cytokines [23]. The study has demonstrated a statistically significant improvement in median PFS in patients receiving sorafenib (5.5 months) compared to patients receiving placebo (2.8 months) (*p* < 0.0001). The magnitude of the benefit obtained with sorafenib versus placebo was similar in patients previously treated with cytokines or cytokine naive patients, in regards to PFS and clinical benefit. No significant difference in terms of OS between patients in both treatment arms (median OS of 19 months in sorafenib arm versus 16 months in placebo arm; *p* = 0.018) has been observed. Based on these results, sorafenib (Nexavar^®^, Bayer HealthCare, Montville, NJ, USA; Onyx Pharmaceuticals, Emeryville, CA, USA) was approved in December 2005 by the FDA and in July 2006 by the EMA for the treatment of advanced RCC.

Axitinib is an oral multi-targeted receptor TKI, which binds to VEGFR-1/2/3, PDGFR, and c-KitR [24,25]. The pivotal phase III trial, the AXIS study, has evaluated axitinib (at a dose of 5 mg twice daily) compared to sorafenib (at a dose of 400 mg twice daily) in 723 RCC patients whose disease had progressed on one previous systemic therapy (cytokines, mTOR inhibitors, and VEGF inhibitors with the exception of axitinib and sorafenib) [26]. The study has demonstrated a statistically significant improvement in median PFS in patients receiving axitinib (6.7 months; 95% CI: 6.3–8.6) compared to patients receiving sorafenib (4.7 months; 95% CI: 4.6–5.6; *p* < 0.0001). Interestingly, the PFS was longer in cytokine-pretreated patients (12 months) than in sunitinib-pre-treated patients (five months) (HR: 0.741; 95% CI: 0.57–0.96; *p* = 0.0107). The ORR was significantly superior in axitinib subgroup (19%) compared to sorafenib subgroup (9%) (*p* = 0.0001). Recently, Motzer et al. have reported updated data about the OS of the AXIS trial [27]. No significant difference in terms of OS between patients in both treatment arms who received the same first-line therapy (median OS of 20 months in axitinib arm versus 19 months in sorafenib arm; *p* = 0.3744) was observed [28]. The OS analysis has allowed that all patients in each arm could continue a third-line treatment after progression on study drug, thus this study design could represent a confounding factor for overall survival results [26]. Based on these results, axitinib (Inlyta^®^, Pfizer Inc., Freiburg, Germany) was approved in January 2012 by the FDA and in August 2013 by the EMA for the treatment of pre-treated advanced RCC.

Cabozantinib is a potent oral multi-targeted TKI, which inhibits VEGFR-1/2/3, MET, c-KitR, FLT-3 and AXL [29]. The pivotal phase III trial, the METEOR study, has evaluated cabozantinib (at dose of 60 mg once daily) versus everolimus an mTOR inhibitor (at dose of 10 mg daily) in advanced RCC 658 patients with progressive disease after VEGFR TKIs [30]. The study has demonstrated a statistically significant improvement in median PFS in patients receiving cabozantinib (7.4 months) compared to patients receiving everolimus (3.8 months). The rate for progression of death while on therapy was higher in the everolimus-treated population (HR 0.58; 95% CI: 0.45 to 0.75; *p* < 0.001). The ORR was higher in those receiving cabozantinib (21%) compared to those on everolimus (5%) (*p* < 0.001). Recently, Choueiri et al. have published the final OS results [31]. The median OS was 21.4 months for patients treated with cabozantinib compared with 16.5 months for those who received everolimus (HR 0.66, 95% CI: 0.53–0.83, *p* < 0.001). Based on this study, cabozantinib (CabometyxTM, Exelixis and Ipsen, Inc.) was approved in April 2016 by the FDA and in July 2016 by the EMA for the treatment of advanced RCC in patients who have received prior anti-VEGF target therapy.

Dovitinib is an oral TKI that inhibits VEGFR-1/2/3, FGFR and PDGFR [32]. In preclinical studies, this agent demonstrated greater antitumor activity when compared to sunitinib and sorafenib [33]. In the pivotal phase III GOLD trial, 284 patients with advanced RCC who received one previous VEGF-targeted therapy and one previous mTOR inhibitor were randomized to receive either dovitinib (at dose of 500 mg orally, five days on and two days off) or sorafenib (at dose of 400 mg orally twice daily) [34]. With a median follow-up of 11.3 months, there was no difference in the mPFS between both arms (3.7 and 3.6 months for dovinitib and sorafenib groups, respectively; HR 0.86, 95% CI: 0.72–1.04; one-sided *p* = 0.063). Dovitinib failed to meet the primary endpoint of PFS versus sorafenib in patients pre-treated and progressive RCC, and, consequently, has not been approved by FDA for the treatment of advanced RCC.

Tivozanib is a potent oral VEGFR-1/2/3 TKI [35]. The TIVO-I study is a phase III trial comparing tivozanib (at dose of 1.5 mg daily, three weeks on and one week off) with sorafenib (400 mg twice daily) in 517 patients who were either untreated or had received cytokines [36]. Overall estimated PFS in the tivozanib arm was 11.9 months versus 9.1 months in the sorafenib arm (*p* = 0.042). Interestingly, PFS was better in patients with no prior treatment, 12.7 months in the tivozanib arm and 9.1 months in the sorafenib arm (*p* = 0.037); ECOG performance status 0, 14.8 months in tivozanib versus 9.1 months in the sorafenib arm (*p* = 0.004); and Memorial Sloan-Kettering Cancer Center favorable prognostic group, 16.7 months in the tivozanib arm versus 10.8 months in the sorafenib arm (*p* = 0.018). The overall ORR was 33% in the tivozanib arm compared with 23% in the sorafenib arm (*p* = 0.014). Importantly, OS analysis showed trend towards longer survival in the sorafenib arm compared with the tivozanib arm (29.3 vs. 28.8 months; *p* = 0.105). It is important to note that 63% (*n* = 162) of patients in the sorafenib arm received next-line therapy and almost all of these patients (156/162) received tivozanib as second-line treatment. Only 13% of patients in the tivozanib arm received subsequent therapy. This study met its primary end point by statistically significantly improving PFS, but did impair OS, a secondary end point. Crossover from sorafenib to tivozanib may have confounded survival. Because of that detrimental survival, the US FDA rejected approval in May 2013.

Lenvatinib is a potent oral multi-targeted TKI, which inhibits VEGFR-1/2/3, FGFR, PDGFRα, RET-R and c-KitR [37]. A phase II clinical trial studied the combination of everolimus (at dose of 5 mg daily) with lenvatinib (at dose of 18 mg daily) versus everolimus (at dose of 10 mg daily) in 153 patients [38]. Median PFS was 14.6 months for lenvatinib plus everolimus and 5.5 months for single-agent everolimus (*p* < 0.001). Patients treated with single-agent lenvatinib had a median PFS of 7.4 months, which was also longer compared with those treated with single-agent everolimus (*p* = 0.048). In an updated OS analysis published by Hutson et al., the median was 25.5 months for lenvatinib plus everolimus, compared with 15.4 months for patients treated with everolimus (*p* = 0.065), which demonstrated a trend towards survival benefit with the experimental combination. Overall, ORR was achieved by 43% of the patients allocated combination regimen compared with 27% assigned lenvatinib (*p* = 0.10) and 6% who received everolimus (*p* < 0.001). Currently, there is no randomized phase III study confirming the results of this study. Nonetheless, based on the positive results from this phase II study, the combination of lenvatinib (Lenvima^®^, Eisai Inc., Tokyo, Japan) and everolimus was recently approved in May 2016 by the FDA and in August 2016 by the EMA in advanced RCC for the treatment of patients pre-treated with one anti-VEGF target therapy.

In Table 1 TKIs targeting tumor angiogenesis used in cc-RCC patients are summarized.

### 1.3. The Need of Imaging Methods for Targeted Therapies

The above-discussed TKIs are now the standard of care for treatment of metastatic RCC. However, they are not side effects-free and are burdened with high costs. Therefore, there is an urgent need in imaging-based methodologies that can early and reliably identify responders and could be used for image-guided optimization and “personalization” of anti-angiogenic regimens [39,40,41,42].

Although tumor size measurements with the response evaluation criteria in solid tumors (RECIST 1.1) have been used for monitoring response to therapy, it is well known that conventional response assessment based on size is not the best parameter in reflecting the clinical effectiveness of cytostatic targeted therapies, as size changes are lag behind actual response [43,44]. In effect, since targeted therapy usually induces only mild lesion shrinkage and metastases can even increase in size while the drug is acting, assessment of response according to RECIST may be misleading [45,46,47,48]. For these reasons, accurate and timely response assessment in the setting of anti-angiogenic therapies remains suboptimal when morphological imaging methods are employed: predicting and monitoring disease progression is a challenge that is unmet by computed tomography (CT) and magnetic resonance imaging MRI.

To prevent premature cessation of efficacious treatment and, conversely, to continue a non-efficacious with toxicity and costly treatment, there is a need to improve imaging response assessment. The new challenge for imaging is to provide timely assessment of disease status, allowing therapies to be tailored to ensure ongoing clinical benefit.

Nowadays, integrated Positron Emission Tomography/CT (PET/CT) is a leading imaging modality used in the diagnosis and staging of many types of solid tumors, thus contributing to clinical decision-making. Whereas conventional imaging techniques can provide information on anatomic abnormalities, PET imaging relies on both molecular biology and in vivo imaging to provide information about the preceding changes in metabolism and function, including glucose metabolism, cell proliferation, cell membrane metabolism, or receptor expression. Deciding when to stop therapy and when to convert to a different TKI is a difficult decision that could be assisted by metabolic evaluation. Furthermore, integrated PET/CT scanner allows correct co-registration and fused imaging of anatomical and functional data. The ability to noninvasively characterize in vivo molecular processes with a relatively fast whole-body scan is the major advantage of this technology.

Various radiotracers are used in different clinical situations even if the possibility of predicting and monitoring response to TKIs therapy has been studied mainly with ^18^F-fluoro-2-deoxy-2-d-glucose (^18^F-FDG).

According to the aforementioned clinical challenges, herein we critically review the key metabolic changes associated with RCC progression and discuss current and future roles in the field of PET/CT for metabolic imaging of cancer and its potential to improve the clinical management of cancer patients treated with TKI.

## 2. Imaging of Glucose Metabolism (^18^F-FDG PET/CT)

### 2.1. Qualitative Assessment

**Figure 2:**
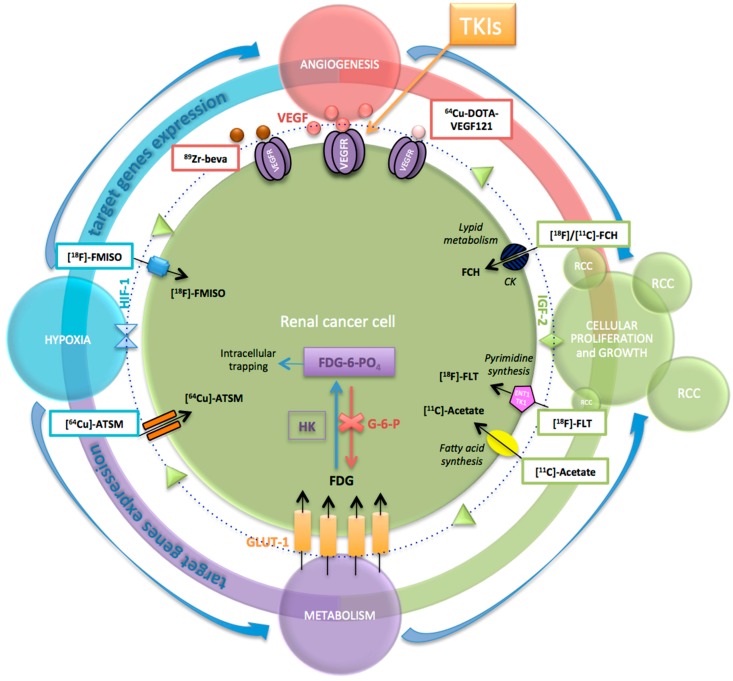
Key steps responsible of cancer disease growth and progression and related main PET radiotracers for biomolecular imaging in RCC. Hypoxia, angiogenesis and glucose metabolism are the main pathways involved in determining the cell growth and proliferation, and they are strictly connected each other like a loop that is self-feeding. As anti-angiogenic agents, TKIs interfere this loop, being able to inhibit tumor proliferation. Their effects can be detectable on PET/CT by change in ^18^F-FDG uptake, the main radiotracer used to date, as a strong indicator of biological response. However, new radiotracers for biometabolic imaging are currently under investigation, which exploit the other pathways involved in the cancer process, including cellular proliferation [^18^F-fluoro-thymidine (^18^F-FLT)], aerobic metabolism (^11^C-acetate), cell membrane synthesis (^11^C-choline, ^18^F-fluorocholine), hypoxia [^18^F-fluoromisonidazole (^18^F-FMISO)], and amino acid transport (^11^C-methionine), as well as the same angiogenic process (^64^Cu-DOTA-VEGF121) and ^89^Zr-bevacizumab. VEGF, Vascular Endothelial Growth Factor; VEGFR, Vascular Endothelial Growth Factor Receptor; TKIs, thyrosin kinase inhibitors; ^89^Zr-beva, 89Zr-bevacizumab; ^64^Cu-DOTA-VEGF121, ^64^Cu-Labeled1,4,7,10-Tetraazacyclododedane-*N*,*N*’,*N*’’,*N*’’’-tetraacetic acid–conjugated vascular endothelial growth factor A isoform 121-gelonin fusion protein; HIF-1, hypoxia-inducible factor 1; ^18^F-FMISO, ^18^F-fluoromisonidazole; ^64^Cu-ATSM, ^64^Cu-diacetyl-bis(N4-methylthiosemicarbazone); GLUT-1, glucose transporter-1; FDG, fluoro-2-deoxy-2-d-glucose; HK, hexokinase II; G-6-P, glucose-6-phosphatase; FDG-6-PO_4_, FDG-6-phosphate; [^18^F]-FLT, ^18^F-fluoro-thymidine, [^18^F]/[^11^C]-FCH, [^18^F]/[^11^C]-fluorocholine; CK, choline kinase.

The use of PET/CT in oncology is rapidly expanding with ^18^F-FDG, the most commonly used radiotracer. The ability of ^18^F-FDG PET/CT to detect cancer is based on increased expression of cellular membrane glucose transporter-1 (GLUT-1) and enhanced hexokinase II enzyme activity within tumor cells. In fact, FDG is a non-physiological radiotracer with a chemical structure similar to that of naturally occurring glucose. It enters cells through the same membrane glucose transporter proteins utilized by glucose, which are commonly overexpressed in cancer cells [49,50]. FDG imaging relies upon Warburg’s observation that increased glycolysis generated adenosine triphosphate is required to meet the metabolic demands of rapidly dividing tumor cells. Membrane glucose transporters, mainly GLUT-1, actively transport FDG into the cell, where hexokinase then converts it into FDG-6-phosphate. As FDG-6-phosphate is not a substrate for further steps in glycolysis, it is trapped in the cell and accumulates correspondingly to the cell’s glucose metabolic activity. Malignant cells exhibit increased FDG accumulation due to increased membrane transporters, increased intracellular hexokinase and low glucose-6-phosphatase [49] (Figure 2).

Nowadays, ^18^F-FDG PET/CT represents the imaging method of greatest impact in the oncology field for the majority of cancer types [51,52]. Unlike for most other malignancies, application of ^18^F-FDG PET/CT is limited for RCC, mainly due to generally low tumor uptake and physiological excretion of ^18^F-FDG from the kidneys and through the urinary system, which may obscure or mask the primary lesions. Although ^18^F-FDG in urological oncology is a challenge, ^18^F-FDG PET/CT was recently demonstrated to be useful when applied to specific indications in selected patients. If its role in diagnosing RCC is conflicting for the previous-mentioned reasons, ^18^F-FDG PET/CT has been more effective in the detection of metastatic disease, particularly when they occurs in rare metastatic sites, thus affecting therapeutic decisions [53]. ^18^F-FDG PET/CT has a high sensitivity and specificity for extra-renal lesions with a sensitivity and specificity that reach more than 84% and 91% in detecting metastases [54]. A recent study by Alongi et al., who evaluated the clinical impact of ^18^F-FDG PET/CT for restaging 104 patients after surgery, demonstrated that the sensitivity and specificity were 74% and 80%, respectively, with impact in the therapeutic management in 43% of cases. Moreover, the authors showed that positive versus negative ^18^F-FDG PET/CT findings were associated with poorer cumulative survival rates over a five-year period (19 vs. 69%, respectively; *p* < 0.05) [55].

One of the most interesting and current applications of ^18^F-FDG PET/CT, in the field of advanced RCC, is monitoring the efficacy of new target therapy such as TKIs treatment, in which ^18^F-FDG PET/CT can play a strong clinical role for evaluating the biological response. Therapy-induced changes in glycolysis occur as early as a few hours following treatment, and surely before any detectable changes in tumor size. Therefore, ^18^F-FDG uptake informs on the suitability of a chosen therapy and allows rapid identification of non-responders who could benefit from alternative interventions. Thus, ^18^F-FDG is increasingly utilized as a non-invasive marker in clinical studies, validating the efficacy of new target therapies for which no reliable biomarkers are currently available.

Among the major groups of targeted drugs currently approved for use against metastatic RCC, sorafenib and sunitinib are two representatives of the former, inhibiting tyrosine kinase VEGFR-2 and PDGFR-β on cancer cell, endothelial cells and pericytes, respectively [56]. Literature reported a correlation between tumor angiogenic activity and ^18^F-FDG uptake, both in vitro and in vivo. Pedersen et al. investigated the association between glucose transporters (GLUTs) and VEGF in 2 human small-cell lung cancer lines, evaluating changes in the expression of GLUTs and VEGF during 12, 18, and 24 h of severe hypoxia in vivo (xenografts) and in vitro (cell cultures). They demonstrated that a co-upregulation of both GLUT-1 and VEGF existed, which suggested a glucose kinetic modulation by angiogenesis-related genes [57]. There is evidence that the upregulation of HIF, induced by hypoxic microenvironment, is able to modulate tumor angiogenesis and increased glucose metabolism [58]. Because expression of GLUTs is also a downstream result of HIF transcriptional activity, it is conceivable that the intensity of ^18^F-FDG uptake on PET by RCC may reflect the variable strength of the HIF signaling pathway and expression of its downstream products, thus being predictive of the effects of inhibitors of this pathway. Airley and Mobasheri analyzed the pathways related to hypoxic regulation of glucose transport, metabolism and angiogenesis and highlighted the link between hypoxia, angiogenesis and glucose transporters in determining the cell growth and proliferation, like a loop that is self-feeding [59] (Figure 2). As anti-angiogenic agents, TKIs interfere with this loop, being able to inhibit tumor proliferation and making evident their effects on PET/CT by change in ^18^F-FDG uptake, a strong indicator of biological response.

Published clinical observations about the use of ^18^F-FDG PET/CT in this setting demonstrated that low ^18^F-FDG uptake before treatment and decreased uptake after two cycles of treatment are associated with better survival. Conversely, higher baseline ^18^F-FDG uptake and higher number of metabolically active lesions correlate with disease aggressiveness, show a lower response rate and are significantly associated with greater risk of disease progression and poorer PFS or OS [54,60,61,62]. In a study involving 77 patients with RCC, Mizuno et al. demonstrated that a higher ^18^F-FDG uptake on PET was associated with elevated tumor levels of phosphorylated-Akt, phosphorylated-S6 protein, aggressive behavior and metastatic potential, early relapse, and shorter OS after radical nephrectomy [63]. Baseline ^18^F-FDG PET/CT yields prognostic significant data, therefore a baseline ^18^F-FDG PET/CT examination is mandatory for the significant information about metabolic activity within lesions that could direct the clinician toward drug selection, and to correctly assess the response to therapy at the post-treatment scan, predicting outcome.

There is little specific data regarding the use of ^18^F-FDG PET/CT for monitoring response assessment in advanced RCC [64]. Despite they are small studies (10–30 patients), their results are encouraging, having found that ^18^F-FDG PET/CT metabolic response may occur within one cycle of therapy and possibly as early as two weeks into treatment with sunitinib or sorafenib as first or second line therapy [65,66]. A preliminary prospective study about the usefulness of PET/CT using ^18^F-FDG in evaluating the early metabolic response to therapy with TKIs in metastatic RCC patients was conducted by Vercellino et al. [65]. In their pilot study, the authors observed a longer PFS in patients with partial metabolic response rather than in patients with stable or progression disease; however, a statistical significance was not reached, presumably due to their small sample size (12 patients). Later, Revheim et al. in a group of 14 patients with metastatic disease treated with sunitinib demonstrated that low ^18^F-FDG uptake before treatment was associated with significantly prolonged survival [60]. Additionally, they confirmed that, after two treatment cycles, a decrease in tracer uptake predicted survival better than lesion shrinkage on CT.

One of the first studies that assessed the advantage of metabolic evaluation in comparison with RECIST was that reported by Lyrdal et al., who studied 10 patients with histologically proven metastatic RCC and evidence of disease progression after previous treatment. Patients underwent PET/CT using ^18^F-FDG at baseline and 1–2 months after sorafenib therapy [67]. Percentage decrease in glycolytic activity were measured on both soft tissue and skeletal lesions; lesion diameter was assessed by using diagnostic CT. Best responders, with a percentage decrease greater than 20% both in soft tissue and skeletal lesions, had significantly better mean OS than patients with least response (18.1 vs. 12.9 months); however, no significant correlation was observed between decrease in ^18^F-FDG uptake and PFS in this study too. A significant 20% decrease in soft tissue lesions diameter was observed on diagnostic CT. The authors demonstrate that ^18^F-FDG PET/CT was more useful than RECIST criteria to evaluate response to TKIs therapy in both soft tissue and skeletal metastases of RCC, since RECIST is limited to soft tissue lesions.

Kakizoe et al. confirmed this point in their study of 48 patients, in which they reported that a decrease in ^18^F-FDG uptake was more pronounced in the abdominal parenchymal organs (24.5%) and less so in the lungs (14.2%), bones (10.4%) and lymph nodes (9.3%). Despite this, the decreased ratio of ^18^F-FDG accumulation in RCC lesions, as assessed one month following initiation of TKI treatment by PET/CT, was not influenced by the site of RCC metastasis [68]. The study suggests that TKIs can be used in the treatment of advanced RCC regardless of the metastatic site, and that FDG PET/CT is a useful method of surveillance to monitor therapeutic response in all lesions.

Ueno et al., in their series of 35 patients with advanced RCC, evaluated the response to TKIs (sunitinib (19 cases) and sorafenib (16 cases)) in terms of tumor size and ^18^F-FDG uptake using PET/CT before and one month after treatment [61]. The mean ratio of SUVmax change and mean ratio in lesion diameter change on CT were obtained to classify patients as good, intermediate or poor responders, and compared with mean PFS and OS for each response group. They observed an average reduction in mean SUVmax from baseline to post-therapy examination (−18%; range −55% to 65%), and a slight reduction in mean lesion diameter (−6%; range −30% to 30%). The authors demonstrated that using a combination of PET (metabolic response) and CT (tumor size response) criteria could predict PFS and OS in these patients. In fact, the Cox-analysis survival of good (lesion diameter sum not increased and SUVmax reduced >20%), intermediate (lesion diameter sum not increased and SUVmax reduced <20%) and poor responders (lesion diameter sum increased or appearance of new lesions) showed statistically significant difference in PFS as well as in OS.

The importance of sequential PET/CT using ^18^F-FDG, performed at various intervals after therapy with sunitinib in patients with newly diagnosed metastatic RCC, as a surrogate marker of response to therapy was investigated by Kayani et al., as a part of a prospective phase II multicenter trial [54]. A total of 44 patients underwent PET/CT at baseline and repeated the examination after the first cycle (at four weeks) and after the third cycle of sunitinib (at 16 weeks). Changes in ^18^F-FDG uptake between the baseline and four weeks, as well as between the baseline and 16 weeks, were calculated and compared with outcome (PFS and OS) data. The multivariate analysis showed that a high ^18^F-FDG uptake demonstrated on PET/CT before treatment, was significantly correlated with shorter OS [hazard ratio (HR): 3.30 and 3.67, respectively]. Moreover, a higher baseline ^18^F-FDG uptake was negatively associated with metabolic response at both four weeks and 16 weeks scans, while 10/12 patients with disease progression on 16-weeks PET-CT had been metabolic responders after one cycle of therapy (that is, four-week scan had no prognostic significance). The authors concluded that ^18^F-FDG PET/CT responses occur in the majority of patients after four weeks of therapy; however, it is not until 16 weeks when the results become prognostically significant.

In the majority of literature studies, a slight-to-moderate reduction in ^18^F-FDG uptake from baseline to post-therapy scan was observed in most patients, while only a minority of patients showed disease progression; a complete metabolic response (i.e., complete normalization of ^18^F-FDG uptake in all lesions in a single patient) was never achievable. Actually, according to Caldarella’s systematic review, in many studies, a good correlation was found between partial metabolic response and PFS and/or OS, with the highest survival rates in patients showing the greatest post-therapeutic reduction ^18^F-FDG uptake [64]. However, the small sample size of the studies may have contributed to this result [58,59,60,65]. Moreover, when analyzing PET/CT in a patient treated with TKIs, it must be remembered that uptake of the tracer by inflammatory cells in the vicinity of sites of responding lesions could also accumulate ^18^F-FDG and can decrease the apparent response to treatment; this could partly explain the apparent low correlation with PFS and OS observed in some studies [46].

### 2.2. Quantitative Assessment

FDG accumulation rate can be semi-quantitatively measured by the maximum standardized uptake value (SUVmax), which is an indirect estimate of the glycolytic activity in the most active pixel within the lesion. Nowadays, this parameter is employed in clinical practice to support the qualitative PET imaging interpretation, achieving the best results in the majority of cancer type also for its prognostic value and treatment guidance [69,70].

Studies performed in patients affected by advanced RCC, showed a negative correlation between baseline SUVmax in the most active lesion and outcome [54,60,71]. Higher baseline SUVmax (≥7.1) and higher number of metabolically active lesions (eight or more) were significantly associated with greater risk of disease progression and poorer PFS or OS. Moreover, patients with higher baseline SUVmax showed a lower response rate than patients with lower baseline SUVmax, even after one cycle of therapy (7.1 in metabolic non-responders vs. 4.4 in metabolic responders). A study that examined 243 metastatic lesions in 26 patients demonstrated a significantly worse median survival time amongst patients in whom the maximum SUV of the primary tumor was >8.8 [72]. The subsequent larger study involving 60 patients also showed a higher mortality of 62.5% in those patients in whom the SUVmax was >10 versus 33.3% in cases where the SUVmax measured 3–5 [54]. These results emphasize the prognostic significance of baseline ^18^F-FDG PET/CT, as the SUVmax is suggestive of aggressive behavior, metastatic potential, early relapse and shorter OS.

SUVmax was used by most authors not only as a predictor of tumor aggressiveness but also as an index to detect eventual changes in metabolic activity from baseline to post-treatment scan. The absolute (SUVdiff) or relative (SUVrel) variation in SUVmax has been used as they reflect the changes in the amount of vital tumor cells induced by therapy. Both Lyrdan et al. and Ueno et al. demonstrated that SUVmax variation, from baseline to post-therapy ^18^F-FDG PET/CT evaluation, correlates with survival [61,67]. In particular, patients with a percentage SUVmax decrease of 20% or more had better median OS (18.1 months) compared to those with a less marked response (12.9).

Recently, several PET parameters were under investigation: SUVpeak, for example, the standardized uptake normalized to lean body mass, or the newer volume based PET parameters such as metabolic tumor volume (MTV) and total lesion glycolysis (TLG). MTV, defined as the volume of tumor tissues with increased ^18^F-FDG uptake, is a novel index in ^18^F-FDG PET and is the least studied factor thus far. It incorporates the dual characteristics of three-dimensional volumetric data and the metabolic activity of tumor. TLG is the product of mean SUV and MTV and was first introduced by Larson et al. to evaluate therapeutic response. It combines the volumetric and metabolic information of ^18^F-FDG PET. Studies have shown the usefulness of TLG for evaluating treatment response in different tumors [73,74,75,76,77].

The use of these volumetric parameters was tested also in metastatic RCC patients, providing an even better correlation with prognosis compared with SUVmax. TLG before treatment demonstrated to be an independent prognostic factor of OS in metastatic cases managed by TKIs. A decrease in TLG and in SUV peak correlated with progression-free and overall survival in 39 patients [78,79]. Hwang SH et al. evaluate the prognostic value of pretreatment MTV and TLG using ^18^F-FDG PET/CT in 56 patients with metastatic RCC after treatment with anti-vascular endothelial growth factor-targeted agents [80]. The authors found that MTV and TLG are independent prognostic factors for predicting PFS and OS in this setting of patients. Furthermore, MTV and TLG could provide additional prognostic information in patients with clinically high-risk metastatic RCC treated with anti-vascular endothelial growth factor-targeted therapies.

These new PET parameters could provide useful information in the early assessment by ^18^F-FDG PET/CT for determining individual patient management strategies. In their study, Faenebo et al. determined whether early changes in the glucose metabolism of metastatic RCC assessed by ^18^F-FDG PET according to the PERCIST 1.0 criteria after 14 and 28 days of treatment with TKIs (sunitinib (18 cases), sorafenib (19 cases), or pazopanib (two cases)) were able to predict OS and PFS in 39 patients [79,81]. They found that early changes in SUVpeak (peak standardized uptake normalized to lean body mass) and total lesion glycolysis after only 14 days of TKI treatment were significantly correlated with both PFS and OS.

**Figure 3:**
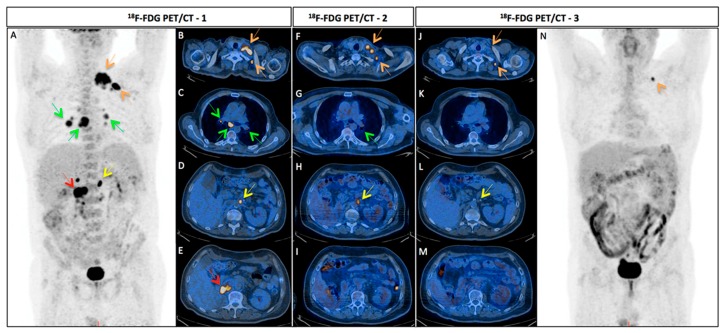
^18^F-FDG PET/CT TKIs therapy evaluation in a 52-year-old man affected by metastatic RCC six months after surgery resection of the right kidney. The ^18^F-FDG PET/CT-1 [MIP (**A**); and axial fused PET/CT (**B**–**E**)] detected several cervical (SUVmax 17.7, orange arrows), mediastinal (SUVmax 13.2, green arrows) and abdominal (SUVmax 16.7, yellow arrow) lymph nodes (**A**–**C**), as well as recurrent disease in right renal loggia (SUVmax 11.9, red arrow) (**E**) at baseline. The ^18^F-FDG PET/CT-2 [axial fused PET/CT (**F**–**I**)] performed after 1 cycle of TKIs demonstrated partial but early disease response with persistent ^18^F-FDG uptake in cervical (SUVmax 12.2-orange arrows), mediastinal (SUVmax 3.4, green arrows) and abdominal (SUVmax 6.7, yellow arrow) lymph nodes (**F**–**H**) and increased metabolism in right renal loggia (**I**), despite only slight reduction in size. In the ^18^F-FDG PET/CT-3 [MIP (**N**); and axial fused PET/CT (**J**–**M**)] performed after six months of TKIs therapy, only a persistent cervical (SUVmax 6.8, orange arrows) lymph node (**J**) was detectable. Other lymph nodes were still recognized on CT examination but they were glucose metabolism-free ((**J**) upper orange arrow; and (**L**) yellow arrow). ^18^F-FDG PET/CT, 18F-fluoro-2-deoxy-2-d-glucose positron emission tomography/computed tomography; TKIs thyrosin kinase inhibitors, RCC, renal cell carcinoma.

Figure 3 shows an example case of a 52-year-old man affected by metastatic RCC, treated with TKIs and evaluated with serial ^18^F-FDG PET/CT. Written informed consent was obtained from the patient.

## 3. Other Radiotracers

Among the various new investigated radiotracers, only few are usable for evaluation of RCC and none is ideal. The type and quality of the images produced by PET/CT are products of a complex interplay between the physics of the isotope, the chemistry of the radiotracer, the biology of the tumor and its surrounding normal tissue, and the timing of image acquisition after injection of radiotracer. Understanding the implications of such a complex interplay demands profound knowledge of physics, chemistry and biology, making the development of new radiotracers extremely challenging.

The new radiotracers for biometabolic imaging, currently under investigation, are expected to further improve the performance of PET in uro-oncology. These new tracers exploit various cellular processes that are altered in malignant cells, including cellular proliferation [^18^F-fluoro-thymidine (^18^F-FLT)], aerobic metabolism (^11^C-acetate), cell membrane synthesis (^11^C-choline, ^18^F-fluorocholine), hypoxia [^18^F-fluoromisonidazole (^18^F-FMISO)], and amino acid transport (^11^C-methionine), as well as the angiogenic process (^64^Cu-DOTA-VEGF121) and ^89^Zr-bevacizumab.

### 3.1. Imaging of Proliferation and Growth

^18^F-Fluorothymidine (^18^F-FLT) is an analog of thymidine. FLT undergoes phosphorylation by thymidine kinase-1 and is trapped inside cells. Its uptake correlates with thymidine kinase-1 activity, and inhibition of cell cycle progression prevents its uptake. Therefore, FLT is a marker of proliferation [82]. Considering the mechanism of action of TKIs, which are expected to decrease the rate of tumor cell proliferation, imaging with FLT has potential for monitoring response to therapy. Excretion of FLT into the urine, as well as its uptake in liver and bone marrow, can potentially limit its usefulness.

Horn et al. compared ^18^F-FLT and ^18^F-FDG for early measurement of response to sunitinib treatment in 20 patients with metastatic RCC and demonstrated that while ^18^F-FLT-PET could be used to identify response as early as one week after the start of treatment, ^18^F-FDG PET was more effective at a later time point of 3–4 weeks, suggesting that inhibition of VEGF signaling with sunitinib exerts an early effect on tumor proliferation, which is then followed by a reduction in tumor metabolism [66].

Liu et al., who studied 16 patients with malignant tumors (seven with RCC), reported a decrease in mean SUV from a pretreatment value of 3.2 to 2.1 during treatment but observed a rebound to 3.1 while patients were off treatment [83]. Average SUVmax similarly decreased from 10 to 6 and increased to 10.1 while off treatment. This finding is consistent with the VEGF receptor tyrosine kinase inhibitor withdrawal flare. Patients with steep initial reduction in SUV also had a steep increase during washout, making interpretation of changes in SUV during treatment challenging.

^18^F/^11^C-Choline-Choline is a new PET tracer, in which uptake may occur via a choline-specific transporter protein and be accelerated during the proliferation of tumor cells. Tissue uptake of radio-labeled choline corresponds to an increase in membrane lipid synthesis [84,85].

Choline, an essential compound of the cell membrane, is actively and passively transported into cells and then phosphorylated by choline kinase. In a further step, it is metabolized to phosphatidylcholine, which is incorporated into the cell membrane. In previous studies, the increased choline uptake in tumor cells was explained mainly by the upregulation of choline kinase and over-expression of choline transporters due to an increased demand of membrane constituents [85,86]. Replacing one of the “natural” carbons of a chemical element of choline with the positron emitting isotope ^11^C creates a substance that is chemically identical to a non-labeled natural choline. However, ^11^C has a short half-life (20 min), limiting its usefulness to institutions with an in-house cyclotron, and its higher Emax positrons (0.96 MeV) reduce spatial resolution. ^11^C-choline is accumulated in the renal cortex and has delayed excretion into the collecting system. Choline can also be labeled with ^18^F, which has a longer half-life but is excreted into the urine in a manner similar to ^18^F-FDG [87].

In vitro and in vivo studies postulated the use of choline analogs as a marker for tumor proliferation [88]. However, this possibility with regard to RCC has not yet been adequately explored. Only two groups have reported the utility of ^18^F-fluoroethylcholine PET compared with ^11^C-acetate PET for staging and monitoring of the response of advanced RCC to therapy [89,90].

^11^C-acetate-Acetate is used in fatty acid biosynthesis by fatty acid synthase, which is highly expressed in RCC and described as an indicator of tumor aggressiveness and poor prognosis [91]. When injected into the blood stream, acetate is rapidly picked up by cells and metabolized into acetyl-CoA. ^11^C-acetate is not eliminated via the efferent urinary tracts, making it a good candidate for evaluation of urothelial carcinoma. Shreve et al. reported on their first experiences with ^11^C-acetate for the diagnosis of various kidney diseases. However, renal cell carcinomas did not show enhanced uptake of ^11^C-acetate in comparison to the surrounding renal parenchyma, but they did have a significantly reduced clearance rate [92].

### 3.2. Imaging of Hypoxia

^18^F-Fluoromisonidazole-^18^F-FMISO is a hypoxia avid tracer that accumulates intracellularly in areas with low O_2_ pressure after diffusion through the cell membrane and reduction by nitro-reductase, in contrast to its washout from non-hypoxic cells [93,94]. Hypoxia is the main condition that induces angiogenesis by HIF that in turn stimulate VEGF and PDGF expression leading an increase of microvascular density, it contributes to resistance to chemotherapy and radiotherapy and it is associated with poor prognosis in many types of cancers. A major limitation of this tracer is the lower contrast between normal and pathological tissue due to its slow clearance from non-hypoxic cells.

Only mild ^18^F-FMISO uptake was found on PET/CT in 17 patients with RCC, where invasive measurements indicated the presence of hypoxia [94]. Hugonnet et al. used ^18^F-FMISO PET/CT in 53 patients with metastatic RCC at the baseline and one month after sunitinib treatment and demonstrated that patients with initially hypoxic targets had shorter PFS than the others, and that target lesions showed decreased ^18^F-FMISO uptake during one month after sunitinib treatment, suggesting that sunitinib decreased the intensity of tumor hypoxia [95].

^64^Cu-diacetyl-bis(*N*4-methylthiosemicarbazone)—an alternative PET tracer for the study of hypoxia—is based on a complex of ^64^Cu with (ATSM) ligands and could be labeled with copper positron emitter radioactive isotopes such as ^64^Cu. ^64^Cu-ATSM is lipophilic and with low molecular weight, so it has a high membrane permeability and therefore a rapid diffusion into cells. The hypoxic specificity of ^64^Cu-ATSM seems to be related to the intracellular reduction of Cu(II) to Cu(I) combined with re-oxidation by intracellular molecular oxygen. Under hypoxic conditions, the unstable Cu(I)–ATSM complex may further dissociate into Cu(I) and ATSM, and Cu(I) ion is trapped into cells, while, in the presence of oxygen, the Cu(I)-ATSM can be re-oxidized into Cu(II)-ATSM, thus allowing efflux from the cell [96].

Bourgeois et al. compared ^18^F-FMISO and ^64^Cu-ATSM concluding that PET imaging has demonstrated a good efficacy in tumor hypoxia imaging with both ^18^F-FMISO and ^64^Cu-ATSM, but ^64^Cu-ATSM appears to be superior in terms of imaging performance [97].

### 3.3. Imaging of Angiogenesis

The imaging of tyrosine kinase VEGFR expression employing TKIs targeting VEGFR in cancer therapy has an important role because the treatment efficacy may be highly variable among different tumor types. Noninvasive imaging to evaluate the level of VEGFR expression can be useful in the choice of the potential more effective treatment.

Several literature studies demonstrated that radiolabeled VEGF isoforms have very high binding affinity and specificity for VEGFRs, but it is still needed to improve the in vivo stability, target affinity and specificity, and pharmacokinetics of these radiopharmaceuticals. Cai et al. labeled several VEGF121 isoforms with ^64^Cu for PET imaging [98]. The limit of these tracers was the high VEGFR-1 expression in the kidney and consequently its high toxicity. Subsequently, ^64^Cu-DOTA-VEGF121 (DEE), a mutant VEGF121 specific for VEGFR-2, has been developed demonstrating lower kidney toxicity. The PET imaging of small-animal revealed rapid, specific, and prominent uptake of this ^64^Cu -DOTA-VEGF121 (DEE) in highly vascularized small tumors with high level of VEGFR-2 expression but significantly lower and sporadic uptake in large tumors with low level of VEGFR-2 expression. The dynamic nature of VEGFR expression during tumor growth was demonstrated; in fact, in the same tumor model, levels of VEGFR expression were different at different sizes and stages [98,99].

Other studies investigated the role of radioisotopes labeled to anti-VEGF human antibodies such as bevacizumab, a drug that acts blocking and neutralizing VEGF. Oosting et al. determined tumor uptake of ^89^Zr-bevacizumab in metastatic RCC patients before and during anti-angiogenic therapy, and concluded that high baseline tumor SUVmax was associated with longer time to progression [100].

Perhaps the ideal radiotracer for predicting response to a medication could be the medication itself. Testing with several labeled TKIs might select the most effective one for the specific patient. Initial investigations with labeled sunitinib have shown high uptake in tumor bearing mice but the clinical implications of this observation are far from clear [101].

## 4. Conclusions

Cancer progression is characterized by extensive metabolic reprogramming. The identification of key pathways and enzymes regulating metabolism in cancer cells provides new opportunities for cancer therapy. This has motivated the development of several specific inhibitors targeting metabolic pathways and their therapeutic evaluation in pre-clinical models or in cancer patients. The translation of these findings into personalized therapy remains a considerable challenge. To this end, the use of PET to non-invasively visualize tumor metabolism is likely to facilitate the implementation of and assessment of new targeted therapies, such as TKIs.

^18^F-FDG PET/CT holds great promise in this setting, providing a whole body biometabolic imaging based on a qualitative examination interpretation supported by a quantitative evaluation with SUVmax and the newer volume-based PET parameters. Using ^18^F-FDG PET/CT before actual treatment may assist in selection of the most effective medication for a specific patient. Pretreatment SUVmax assessed by ^18^F-FDG PET/CT can provide helpful information for clinical decision-making as it can serve as a useful prognostic marker for patients with advanced RCC. High SUVmax in patients with primary RCC is suggested with correlate with a high likelihood of metastasis, and FDG accumulation may be useful in estimating patient’s survival.

Moreover, predicting prognosis and response to targeted therapy is a great challenge and a great opportunity in modern nuclear medicine. ^18^F-FDG PET/CT can guide the clinician toward initiating, continuing, stopping or modifying the agent used, since marked changes in tumor diameter are not expected with many therapies. Modern targeted therapy with TKIs usually induces only mild lesion shrinkage making the assessment of response according to RECIST insufficient. More prospective and larger studies about the role of ^18^F-FDG PET/CT in TKIs therapy response assessment are still needed to confirm these interesting results.

While ^18^F-FDG remains the gold standard for imaging of glycolytic tumors, new PET radiotracers for functional imaging are currently under investigation, which explores alternative metabolic pathways for growth, as markers of hypoxia, proliferation and/or angiogenesis, and expected to further improve the performance of PET in uro-oncology. However, they require of further investigation.

## Figures and Tables

**Table 1:** Tyrosine kinase inhibitors targeting tumor angiogenesis currently or recently approved in clear cell renal cancer patients.

Drug	Mechanism of Action	Pivotal Study	Comparator	Line of Treatment	Study Design	EMA Approval
Sunitinib	VEGFR-1/2/3, PDGFRα/β, c-KitR, FLT-3, CSF1-R and RET-R inhibitor	Motzer RJ, NEJM 2007	IFN-α	First-line in MSKCC good/int/high risk patients and after first-line treatment with cytokines	Phase III	Yes
		Gore ME, Lancet Oncol 2009				
Pazopanib	VEGFR-1/2/3, PDGFRα/β and c-KitR inhibitor	Sternberg CN, JCO 2010	Placebo	First-line in MSKCC good/int risk patients and after first-line treatment with cytokines	Phase III	Yes
		Sternberg CN, Eur J Cancer 2013				
Sorafenib	BRAF, VEGFR-2, PDGFR, FLT-3 and c-KitR inhibitor	TARGET study	Placebo	First-line in MSKCC good/int risk patients and after first-line treatment with cytokines or VEGF/VEGFR inhibitors	Phase III	Yes
		Escudier B, NEJM 2007				
		INTORSECT study				
		Hutson TE, JCO 2014				
Axitinib	VEGFR-1/2/3 inhibitor	AXIS study	Sorafenib	After first-line treatment with cytokines or VEGF/VEGFR inhibitors	Phase III	Yes
		RINI B, Lancet 2011				
Cabozantinib	VEGFR-1/2/3, MET, cKitR, FLT-3 and AXL inhibitor	METEOR study	Everolimus	After first-line treatment with VEGF/VEGFR inhibitors	Phase III	Yes
		Choueri NEJM 2015				
Dovitinib	VEGFR-1/2/3, FGFR and PDGFR inhibitor	GOLD study	Sorafenib	-	Phase III	No
		Motzer RJ, Lancet Oncol 2014				
Tivozanib	VEGFR-1/2/3 inhibitor	TIVO-1 study	Sorafenib	-	Phase III	No
		Motzer RJ, JCO 2013				
Lenvatinib	VEGFR-1/3, FGFR1–4, PDGFRα, RET-R and c-KitR inhibitor	Study 205	Everolimus	-	Phase II	Yes
		Motzer RJ, Lancet Oncol 2015				

VEGFR, Vascular Endothelial Growth Factor Receptor; PDGFR, Platelet Derived Growth Factor Receptor; c-KitR, c-Kit Receptor; FLT-3, FMS-like Tyrosine Kinase 3; CSF1-R, Colony Stimulating Factor 1 Receptor; RET-R, REarranged during Transfection Receptor; IFN, Interferon; MSKCC, Memorial Sloan Kettering Cancer Center; BRAF, B-Raf Receptor; MET, Mesenchymal-Epithelial Transition Factor or Hepatocyte Growth Factor Receptor; AXL, AXL Receptor; FGFR, Fibroblast Growth Factor Receptor.
